# Experimental realization of neutron helical waves

**DOI:** 10.1126/sciadv.add2002

**Published:** 2022-11-18

**Authors:** Dusan Sarenac, Melissa E. Henderson, Huseyin Ekinci, Charles W. Clark, David G. Cory, Lisa DeBeer-Schmitt, Michael G. Huber, Connor Kapahi, Dmitry A. Pushin

**Affiliations:** ^1^ Institute for Quantum Computing, University of Waterloo, Waterloo, ON N2L3G1, Canada.; ^2^ Department of Physics and Astronomy, University of Waterloo, Waterloo, ON N2L3G1, Canada.; ^3^ Joint Quantum Institute, National Institute of Standards and Technology and University of Maryland, College Park, MD 20742, USA.; ^4^ Department of Chemistry, University of Waterloo, Waterloo, ON N2L3G1, Canada.; ^5^ Neutron Scattering Division, Oak Ridge National Laboratory, Oak Ridge, TN 37831, USA.; ^6^ National Institute of Standards and Technology, Gaithersburg, MD 20899, USA.

## Abstract

Methods of preparation and analysis of structured waves of light, electrons, and atoms have been advancing rapidly. Despite the proven power of neutrons for material characterization and studies of fundamental physics, neutron science has not been able to fully integrate these techniques because of small transverse coherence lengths, the relatively poor resolution of spatial detectors, and low fluence rates. Here, we demonstrate methods that are practical with the existing technologies and show the experimental achievement of neutron helical wavefronts that carry well-defined orbital angular momentum values. We discuss possible applications and extensions to spin-orbit correlations and material characterization techniques.

## Teaser

Arrays of fork dislocation phase gratings enable the tuning of neutron vortex beams that carry orbital angular momentum.

## INTRODUCTION

Neutrons play a vital role in the characterization of materials and the experimental verification of fundamental physics (*1*, *2*). They are distinguished as a unique probe by their nanometer-sized wavelengths, magnetic sensitivity, and penetrating abilities stemming from electrical neutrality. Further versatility is enabled by the coupling of neutron’s internal and spatial degrees of freedom (*3*) and advances in neutron interferometry techniques that exploit the phase degree of freedom of the neutron wave function (*4*, *5*).

Structured wave functions of light, electrons, and atoms have become widely used scientific tools (*6*–*13*). A general class of structured wave functions is indexed by orbital angular momentum (OAM) (*14*–*18*), in which the wave function varies as *e*^*i*𝓁ϕ^, where 𝓁 is the OAM value and the angle ϕ describes the azimuth around the propagation vector. The OAM states have a “helical” or “twisted” wavefront, and they have been shown to manifest unique sets of selection rules and scattering cross sections when interacting with matter (*19*–*23*).

It is straightforward to impress OAM upon light with refractive and diffractive optics. However, the full extension of OAM techniques into neutron science has been complicated by several factors. First, the neutron index of refraction of common materials is on the order of *n* ≈ 1 − 10^−5^. Thus, the basic optimal element, a lens, is not practical in neutron setups. Second, the spatial coherence of neutron beams is, at most, a few micrometers, as it is typically set by the neutron wavelength, the aperture size, and the distance from the aperture to the sample (*24*). The imaging of fine details of diffraction is further hindered by the spatial resolution of neutron cameras that are, at best, a fraction of a millimeter. Last, at powerful research reactors, the fluence rate of thermal neutrons at monochromatic [polychromatic] beamlines reaches 10^5^ [10^7^] neutrons/(cm^2^ s). For comparison, note that a typical 1-mW red laser with a common spot size area in the range of square millimeter to square centimeter emits more than 10^15^ photon/s.

Several experimental demonstrations with neutron OAM have been presented (*25*–*27*), reporting the first manipulation of the average neutron OAM value using interferometric measurements of relative phase shifts. However, the creation of a neutron state with helical wavefronts dominated by a single OAM value remained elusive. The prospect of a wide range of notable applications ushered forth diverse proposals and a lively discussion concerning the possibilities of generating and measuring quantized neutron OAM given the technical challenges (*28*–*34*).

Here, we demonstrate a holographic approach to tuning of neutron OAM. We use microfabricated arrays of millions of diffraction gratings with critical dimensions comparable to neutron coherence lengths. The arrays can be laid out on the square centimeter areas typical of usual neutron scattering targets. This suggests possibilities for the direct integration of other structured wave techniques, such as the generation of Airy and Bessel beams (*35*–*37*), into neutron sciences. Furthermore, we discuss the applications toward characterization of materials, helical neutron interactions, and spin-orbit correlations.

Phase gratings with *q*-fold fork dislocations are a standard tool in optics that produce photons with an OAM value of 𝓁 = *mħq* at the *m*-th order of diffraction (*38*). This requires that the transverse coherence length of the light beam be at least comparable to the dimensions of the fork dislocation.

Neutron beams have transverse coherence lengths of micrometers and fluence rates of 10^5^ to 10^7^ neutrons/(cm^2^ s). Observing the neutron signal from a single micrometer-sized target is impractical. However, we can multiply the signal using an *N* × *N* array of micrometer-sized fork dislocation gratings. When considering an array, it is important that the overall array size is much smaller than the diffraction signal of interest and that the separation distance between the individual gratings is large enough so that it does not induce an observable diffraction order.

**Fig. 1.**
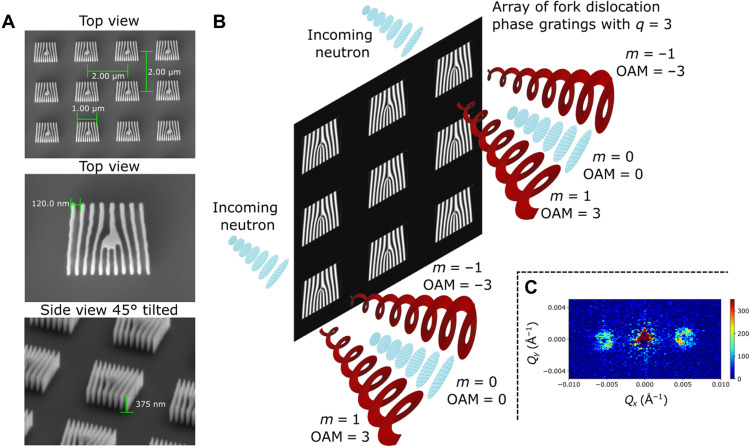
Holographic approach to generating neutron helical wavefronts that carry well-defined OAM. (**A**) SEM images characterizing the array of fork dislocation phase gratings used to generate the neutron helical wavefronts. The arrays covered an area of 0.5 cm by 0.5 cm and consisted of 6,250,000 individual 1 μm–by–1 μm fork dislocation phase gratings that had a period of 120 nm, had a height of 500 nm, and were separated by 1 μm on each sides. Three arrays with topological charges of *q* = 0 (standard grating profile), *q* = 3 (shown here), and *q* = 7 were used in the experiment. (**B**) Each phase grating generates a diffraction spectra consisting of diffraction orders (*m*) that carry a well-defined OAM value of ℓ = *mħq*. (**C**) Intensity in the far field is the sum over the signal from all of the individual fork dislocation phase gratings. Shown is an example of the collected small-angle neutron scattering (SANS) data.

We have fabricated these arrays with *N* = 2500 on silicon substrates using electron beam lithography. Figure 1A shows scanning electron microscope (SEM) images of the fork dislocation phase gratings with *q* = 3. By construction, the spatial dimensions of the individual gratings are comparable to the transverse coherence length of our neutron beam. The use of such an array increases the neutron intensity by *N*^2^ in a given *m* > 0 diffraction order in the far field (see Fig. 1B). The individual diffraction orders in the presented intensity profiles (see Fig. 1C), which span an area of ≈10 cm by 10 cm, were taken over a period of ≈40 min and consisted of the signal from 6,250,000 individual fork dislocation phase gratings.

To characterize the generated OAM states, we can map out the momentum distribution. Small-angle neutron scattering (SANS) beamlines provide several advantages as they map the spatial profiles in the far field, where the observed intensity distribution is directly determined by the Fourier transform of the outgoing neutron wave function. Having access to the far field enables the use of holographic techniques that have been developed for optical structured waves (*38*). Another advantage is the relatively large flux and the accessibility to a wide range of wavelengths. Last, it is the typical setup used in material characterization techniques including the contemporary techniques analyzing skyrmion and topological geometries (*39*, *40*). Straightforward extensions follow for incorporating the characterization of materials and performing experiments with helical neutron interactions.

A simple map for modeling the action of a binary phase grating with a fork dislocation can be expressed as

$$\psi_{\text{in}}\to \psi_{\text{in}}[\text{cos}\;(\frac{\alpha }{2})+\text{sin}\;(\frac{\alpha }{2})\sum_{m}\frac{2}{m\pi }e^{i\frac{2\pi \mathrm{mx}}{p}}e^{\mathrm{imq}\phi }]$$ (1)

where *x* (ϕ) is the Cartesian (azimuthal) coordinate; *p* is the grating period; *m* = … −3, −1,1,3… are the nonzero diffraction orders; α is the induced phase by the height of the grating grooves (see section SA); and the incoming wave function ψ_in_ is typically taken to be a Gaussian profile for convenience. The far field is typically defined to be the distance at which Fraunhofer diffraction is valid. In this regime, the diffraction orders are spatially separated, so here, we can consider the *m* terms independently along their respective propagation directions. We thus obtain well-defined OAM states in the form of ψ_in_*e*^*imq*ϕ^. Note that the OAM is therefore only well defined in the paraxial approximation. The full analysis of the evolution of these states is presented in (*41*, *42*).

With equal transverse coherence lengths σ*_x_* = σ*_y_* = σ_⊥_, we can make use of the cylindrical symmetry to describe the transverse wave function in terms of solutions to the two-dimensional (2D) harmonic oscillator (*32*)

$$\psi_{\ell ,n_{r}}(r,\phi )=N{\left(\frac{r}{\sigma_{⊥}}\right)}^{|\ell |}e^{-\frac{r^{2}}{2\sigma_{⊥}^{2}}}L_{n_{r}}^{|\ell |}(\frac{r^{2}}{\sigma_{⊥}^{2}})e^{i\ell \phi }$$ (2)

where $N=\frac{1}{\sigma_{⊥}}\sqrt{\frac{n_{r}!}{\pi (n_{r}+|\ell |)!}}$ is the normalization constant and *n_r_* ∈ (0,1,2…), 𝓁 ∈ (0, ±1, ±2…), and $L_{n_{r}}^{|\ell |}(r^{2}/\sigma_{⊥}^{2})$ are the associated Laguerre polynomials. The corresponding neutron energy is

$$E=\hbar \omega_{⊥}(2n_{r}+|\ell |+1)$$ (3)

where $\omega_{⊥}^{2}=\hbar /(2M\sigma_{⊥}^{2})$ and *M* is the mass of the neutron. Each diffraction order *m* of the fork dislocation phase grating is in a definite state of OAM

$$\psi =\sum_{n_{r}}\psi_{\ell =\mathrm{mq},n_{r}}$$ (4)

Considering *n_r_* = 0 dominant term (*32*) of the first diffraction order, we can determine that the azimuthally integrated intensity

$$\int_{0}^{2\pi }{|\psi_{q,0}(r_{0},\phi_{0})|}^{2}d\phi$$ (5)

peaks at

$$r_{0}=\sigma_{⊥}\sqrt{q}$$ (6)

## RESULTS

We fabricated three arrays of fork dislocation phase gratings on Si wafers, with topological charge of *q* = 0, 3, and 7. Each array covered an area of 0.5 cm by 0.5 cm and consisted of 6,250,000 individual 1 μm–by–1 μm fork dislocation phase gratings, where each one had a period of 120 nm, had a height of 500 nm, and was separated by 1 μm on each side from the other fork dislocation phase gratings. The full consideration for the parameters is given in section SA and the fab procedure in section SB.

**Fig. 2.**
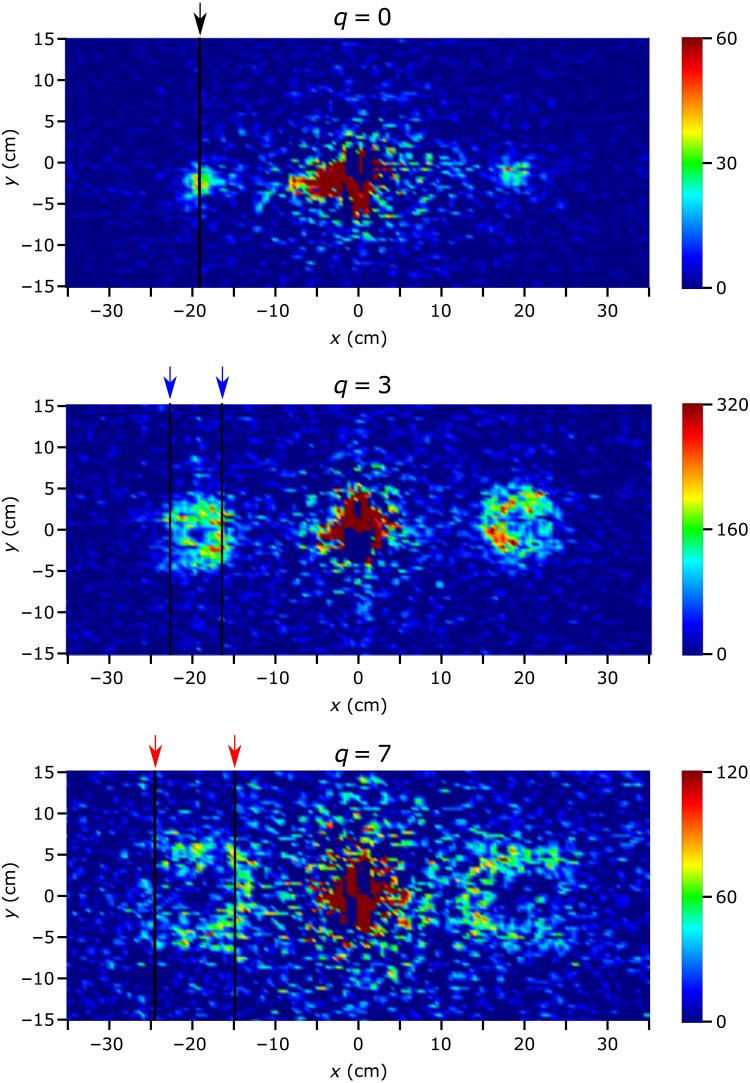
SANS data collected for the three arrays of fork dislocation phase gratings. (**Top**), *q* = 0, (**middle**), *q* = 3, (**bottom**) *q* = 7. The *m* = −1, 0, and 1 diffraction orders are visible, and the corresponding doughnut profiles induced by the helical wavefronts (see Fig. 1B) can be observed in the bottom two profiles. Because of the relatively high intensity of the direct beam, the range of the color bar is limited to emphasize the diffraction order features. The vertical gridlines and the arrows above the plots indicate the location of the theoretical intensity peaks of the *m* = −1 orders. The azimuthally integrated intensity profiles across the diffraction orders, as well as the corresponding simulated profiles, are shown in Fig. 3.

The observed SANS data for the arrays of fork dislocation phase gratings are shown in Fig. 2. The measurement time was 60 min for *q* = 0, 40 min for *q* = 3, and 60 min for *q* = 7. With the phase-grating period of *p* = 120 nm, the angle of divergence of the first diffraction order is θ ≈ λ/*p* ≈ 0.01 rad, which corresponds to $Q_{x}=2\pi /p=0.00525\;{\overset{{}^{\circ}}{A}}^{-1}$ on the SANS images (see Fig. 1C). In our particular setup, this corresponds to a spatial distance of *x* ≈ 19 cm on the camera, as shown in Fig. 2. Good agreement is found with the observed location of the peaks.

**Fig. 3.**
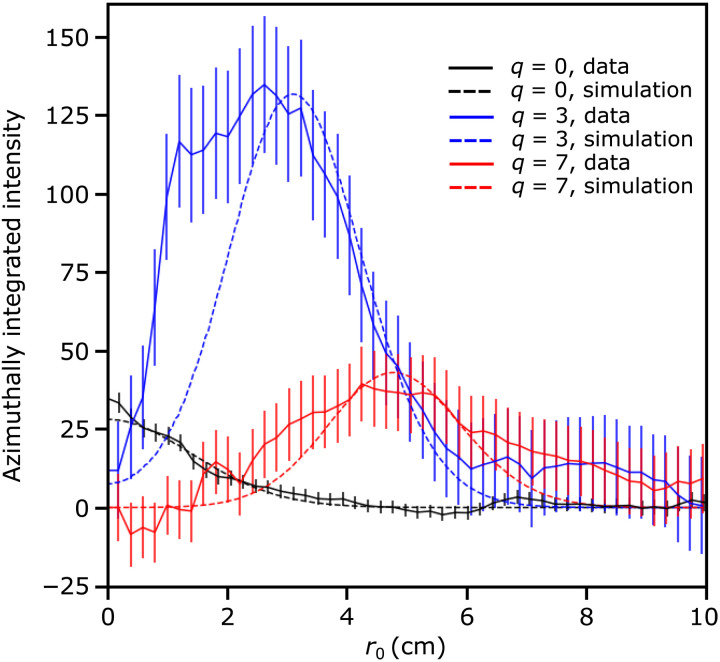
The azimuthally integrated intensity profiles centered on the *m* = −1 diffraction orders of the data presented in Fig. 2. The simulated profiles take into account the wavelength distribution, beam divergence, propagation distance, and array size, which are common to all three cases. The amplitude in each case was scaled to match the observed data. Note that for the *q* = 0 data, a large magnet apparatus was present in the beamline, which greatly reduced the observed intensity. Good agreement is found between the simulated and observed profiles.

To quantify the doughnut profiles, we can analyze the azimuthally integrated intensity (Eq. 5) centered on the first diffraction orders. Figure 3 shows the comparison between observed and simulated intensity. For the simulated profiles, only the amplitude was varied in each case to match the observed amplitude. Wavelength distribution, beam divergence, propagation distance, and array size are common to all three profiles. Note that because each wavelength has a distinct diffraction angle, the observed signal at the first diffraction orders is the incoherent sum of the translated signals from each wavelength. For the *q* = 0 data, a large magnet apparatus was present in the beamline, which greatly reduced the observed intensity, and therefore, the peak appears relatively smaller than expected. Good agreement is observed between the simulated and observed profiles.

## DISCUSSION

We have described and experimentally demonstrated a method to generate and characterize neutron helical waves that are dominated by a well-defined OAM value. The experimental demonstration consisted of fabricating a 2D array of fork dislocation phase gratings, with a pattern area of 0.5 cm by 0.5 cm that amounts to 6,250,000 individual fork dislocation phase gratings contributing to the observed signal. *q* = 0, 3, and 7 topologies were tested, and the obtained doughnut-shaped intensity distributions are in good agreement with the theory.

The presented method opens the door for the implementation of other structured wave techniques with neutrons, such as the generation of “self-accelerating” Airy beams (*36*, *43*) and the “nondiffractive” Bessel beams (*37*, *44*). These beams have a “self-healing” property, as they can reform after being partially obstructed. Considering an array of phase gratings, the Airy beams would be generated through the addition of a cubic phase gradient, while the Bessel beams would be generated through the addition of a radial phase gradient.

The convenient integration with a SANS beamline provides access to the far field where the helical beams with specific OAM values are separated in the form of diffraction orders. This enables studies of interactions between neutron’s helical degree of freedom and scattering from materials. For example, placing the array of fork dislocation phase gratings before a topological sample (*39*, *40*) and postselecting the analysis on individual diffraction orders allow for direct study of scattering properties from neutron helical waves.

Another avenue of exploration that is made possible is the experimental investigation of neutron selection rules. For example, this may be achieved through the addition of ^3^He spin filters. The absorption of neutrons by nuclear spin-polarized ^3^He is strongly dependent on the spin orientation of the neutron because of the conservation of spin angular momentum. Our method allows for the direct tests at a SANS beamline through the characterization of the diffraction peaks after the postselection via ^3^He cell polarizers.

## MATERIALS AND METHODS

SANS measurements were performed on the GP-SANS beamline at the High Flux Isotope Reactor at Oak Ridge National Laboratory (*45*). The arrays of fork dislocation phase gratings were placed inside a rotation mount that was then affixed to the end of the sample aperture holder. The phase gratings were placed 17.8 m away from a 20-mm-diameter source aperture. A 4-mm-diameter sample aperture was placed right in front of the sample. The distance from the phase grating to the camera was 19 m, and the camera size spans an area of ≈1 m^2^, with each pixel being ≈5.5 mm by 4.1 mm in size. The wavelength distribution was triangular with Δλ/λ ≈0.13 and a central wavelength of 12 Å. The resulting beam divergence is ≈0.675 mrad, the transverse coherence of the neutron wave packet σ_⊥_ ≈ λ*L*_1_/*s* ≈ 1 μm, and the SD of the resolution distribution was estimated as σ*_Q_* = 0.00016 Å^−1^.

The neutron wavelength is selected by a turbine-like velocity selector that has helical blades, which allow specified neutrons with correct velocity (and, thus, wavelength) through it. The Δλ/λ is determined by the angle at which the velocity selector is positioned with respect to the beam. Note that because the wavelengths were selected with a velocity selector, we do not get λ/2 and λ/4 contributions that are present when a monochromator is used.

Scattering images were collected for the three arrays of fork dislocation phase gratings in the beam, where the instrument configuration remained fixed. An empty beam scan without a sample and a background scan for a plain Si wafer of equivalent size and thickness were collected. These measurements were used to take into account the beam size, total neutron monitor counts, background and plain wafer scattering, and sample/plain wafer transmission, which would otherwise contribute to losses in intensity and increased background scattering noise levels in the images.

## Supplementary Materials

### This PDF file includes:

Sections SA and SB Figs. S1 to S3
