# Supplementary material for: Experimental realization of neutron helical waves
Source: Sci Adv. 2022 Nov 18;8(46):eadd2002. doi: 10.1126/sciadv.add2002 (PMC9674294; doi:10.1126/sciadv.add2002)
Supplement: Supplementary file 1 — Sections SA and SB Figs. S1 to S3 [file sciadv.add2002_sm.pdf]

Science Advances
AAAS

Supplementary Materials for

Experimental realization of neutron helical waves

Dusan Sarenac *et al.*

Corresponding author: Dusan Sarenac, dsarenac@uwaterloo.ca; Dmitry A. Pushin, dmitry.pushin@uwaterloo.ca

Sci. Adv. **8**, eadd2002 (2022)
DOI: 10.1126/sciadv.add2002

This PDF file includes:

Sections SA and SB
Figs. S1 to S3

SUPPLEMENTARY MATERIAL

A. Fork Array Parameters

A 50% duty cycle phase-grating with a fork dislocation has the following profile:

$$F(x) = \frac{\alpha}{2}\text{sign}\left(\sin\left[\frac{2\pi}{p}x + q\phi\right]\right), \tag{8}$$

where p is grating period, q is the topological charge, x is the Cartesian coordinate, and ϕ is the azimuthal coordinate. The groove height h sets the phase shift that is induced by each grating groove: $\alpha = -Nb_c\lambda h$ where Nb_c is the coherent scattering length density of the grating material and λ is the neutron wavelength. As per Eq. 1 we can see that α determines the relative amplitudes of the diffraction orders. For example, for $\alpha = \pi$ the zeroth order is suppressed while for $\alpha = \pi/2$ there is an equal amount of zeroth order and the higher orders. Note that the fabrication of the small periods currently limits us to small α, and hence we will not be considering these effects. The only consideration is given to minimizing the acquisition time by maximizing the height h to increase the number of neutrons in the first diffraction order.

The resulting profile for $q = 3$ is shown in Fig. 1a. The period p determines the angle of propagation of the diffraction orders, and hence it needs to be set by the requirements of the given beamline. The angle of propagation of the first diffraction order is:

$$\theta = \sin^{-1}\left(\frac{\lambda}{p}\right) \approx \frac{\lambda}{p} \approx \frac{\lambda Q_G}{2\pi} \tag{9}$$

where $Q_G = 2\pi/p$ is the scattering vector of the gratings.

The topological charge q sets the OAM values carried by the diffraction orders. As depicted on Fig. 1a, q is the difference between the number of periods along the grating direction above the origin when compared to the number below the origin.

It is important to note that the fabrication challenges of the central region of the ideal fork dislocation phase-grating are typically overcome through the omission of the central region. Accordingly, a flat circular profile with diameter $\approx$ 200 nm was intentionally imposed in our designs, which can be observed in the SEM figures. This is a common practice in optical OAM techniques given that the effects of such a feature are negligible in diffraction as the beam diverges away from the center.

Lastly the purpose of creating an array of these structures is two-fold. The first is that by creating the array with identical copies we are able to increase the signal to measurable amounts. Note that the spacing between the individual fork dislocation phase-gratings is 1 μm over the 0.5 cm by 0.5 cm area, whereas the diameter of the ring in the first diffraction order is several centimeters. Hence the measured signal is the integral over the positions of the individual gratings whose separation distance and size is much smaller than the signal of interest. The $q = 3$ images shown in Fig. 2 were taken over 40 min and consist of the signal from millions of fork dislocation phase-gratings. Therefore, it is not practical to measure the signal of a single such grating. The second advantage is that future studies with materials are conveniently integratable with this design. A topological material typically possesses an array of topologies and hence it is desirable to create a tool with similar properties for more specific characterization.

B. Fork Array Microfabrication and Characterization

The target parameters for the fork dislocation phase-gratings were: period: $p = 120$ nm for all three arrays, groove height of $h = 450, 500, 400$ nm for q=0, 3, 7, and inner region diameter 200 nm for both $q = 3$ and $q = 7$. These parameters were experimentally optimized to ensure that we obtain high quality and robust structures. The SEM characterization images for q=0, 3, and 7 are shown in Fig. S1, Fig. S2, and Fig. S3 respectively.

Double-side polished, intrinsic, 2 inch diameter (100) silicon wafers were used to fabricate the arrays of fork dislocation phase-gratings. Electron beam lithography (EBL) was employed to pattern the high performance positive EB resists (ZEP520A, $\approx$ 80 nm). The e-beam exposure was carried out with a JEOL JBX-6300FS EBL system operating at 100 kV and 2 nA beam current. The e-beam dosage was 250 μC/cm^2. After the e-beam exposure, the sample was processed in the developer ZED-N50 for 90 s, and then immersed in isopropyl alcohol (IPA) for 60 s followed by a pure nitrogen dry. As a hard mask during plasma etching of Si, Cr metal (20 nm) was e-beam evaporated and lifted-off in a heated PG Remover. A pseudo-Bosch recipe was adopted to achieve a vertical sidewall etch profile. The samples were etched in an Oxford PlasmaLab ICP-380 inductively coupled plasma reactive ion etching (ICP-RIE) system, which provides high-density plasma with independently controlled system parameters. The recipe includes rf: 100 W, ICP:1200 W, C_4F_8: 25 SCCM, SF_6: 15 SCCM, pressure: 1.333 Pa, temperature: 20°C. After fabricating the array of fork dislocation phase-gratings, the remaining Cr etch mask was removed via plasma etching.

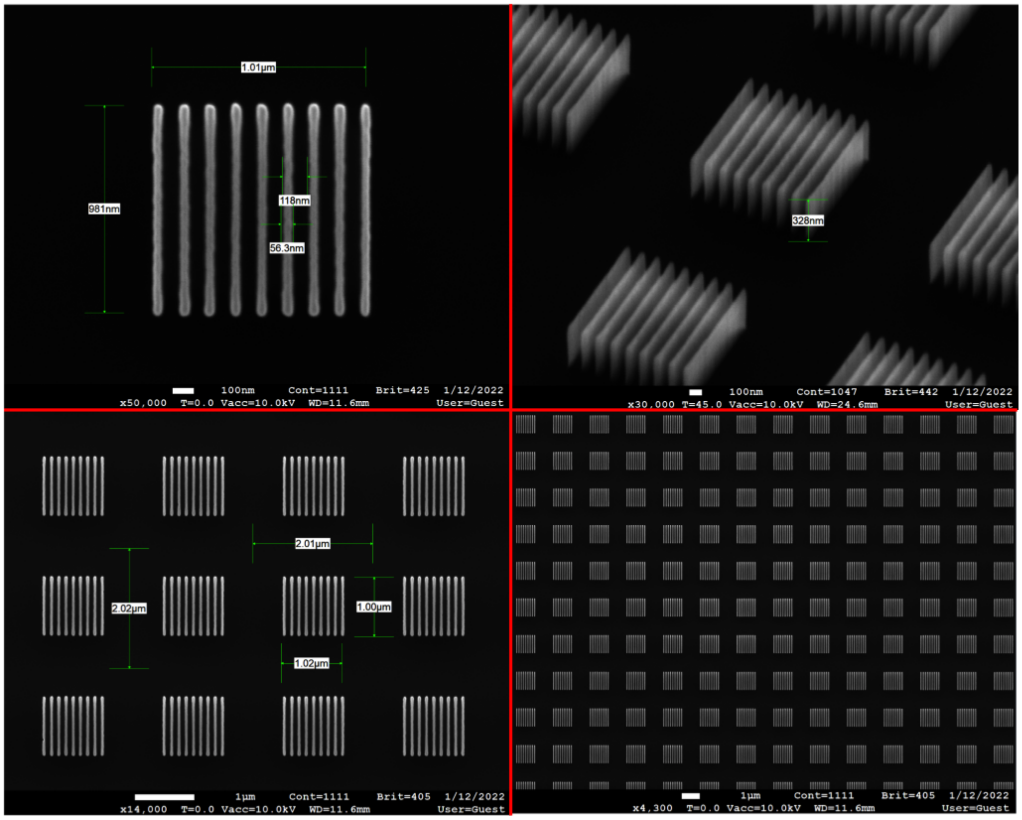

FIG. S1. **SEM characterization of the 2D fork dislocation phase-grating array with topology q =0, which is the standard grating profile.** Shown images are top view except the top right image that is at the 45° tilt. The array covered a 0.5 cm by 0.5 cm area and consisted of 6,250,000 individual 1 μm by 1 μm phase-gratings, where each grating possessed a period of 120 nm, height 450 nm, and was separated by 1 μm on each side from the other gratings.

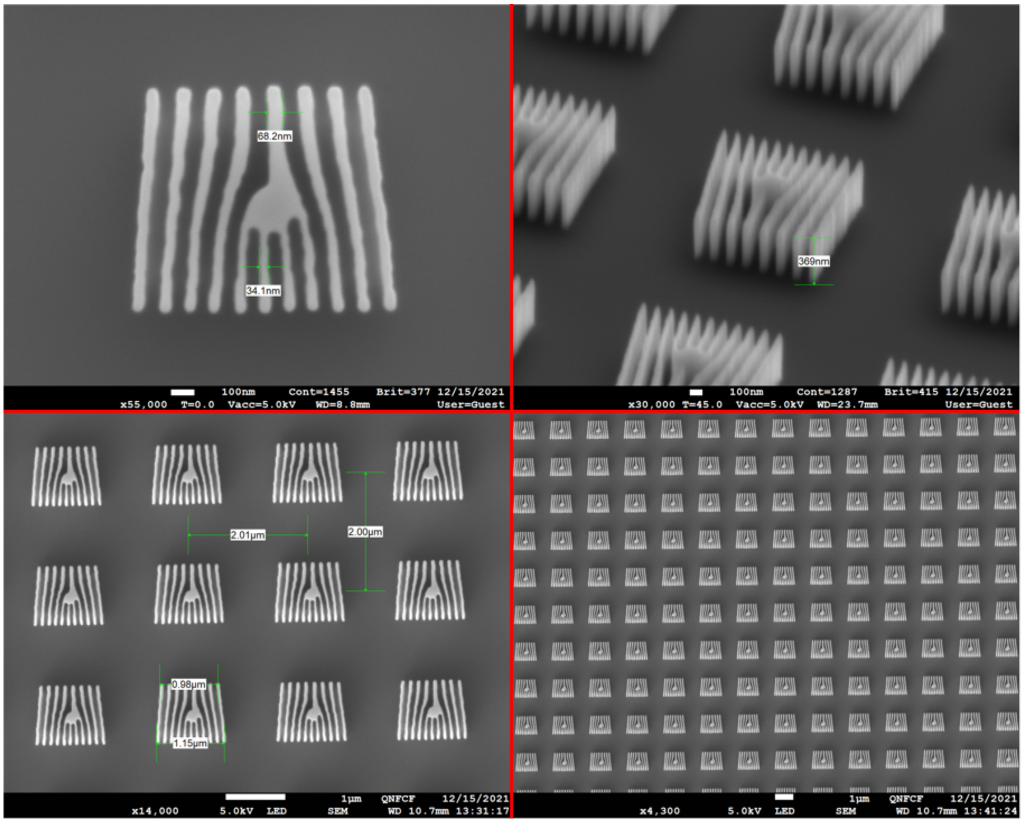

FIG. S2. **SEM characterization of the 2D fork dislocation phase-grating array with topology q =3**. Shown images are top view except the top right image that is at the 45° tilt. The array covered a 0.5 cm by 0.5 cm area and consisted of 6,250,000 individual 1 μm by 1 μm fork dislocation phase-gratings, where each grating possessed a period of 120 nm, height 500 nm, and was separated by 1 μm on each side from the other gratings.

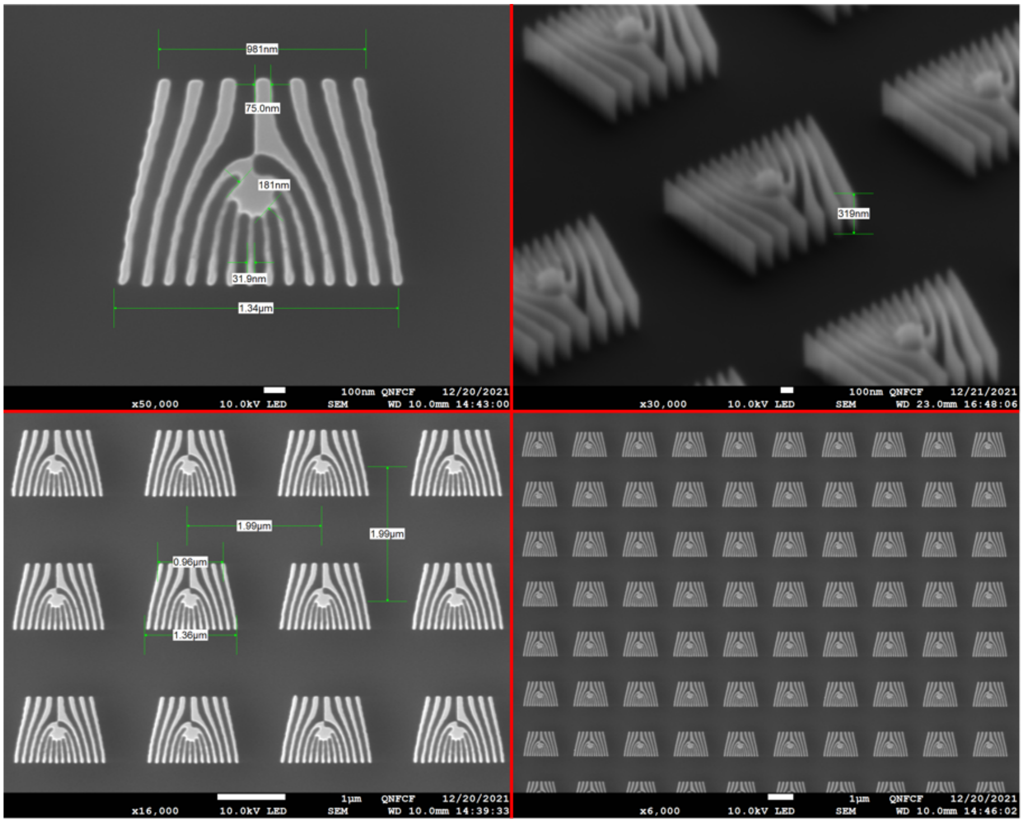

FIG. S3. **SEM characterization of the 2D fork dislocation phase-grating array with topology q =7**. Shown images are top view except the top right image that is at the 45° tilt. The array covered a 0.5 cm by 0.5 cm area and consisted of 6,250,000 individual 1 μm by 1 μm fork dislocation phase-gratings, where each grating possessed a period of 120 nm, height 400 nm, and was separated by 1 μm on each side from the other gratings.
